# Physical Characterization of Gemini Surfactant-Based Synthetic Vectors for the Delivery of Linear Covalently Closed (LCC) DNA Ministrings

**DOI:** 10.1371/journal.pone.0142875

**Published:** 2015-11-12

**Authors:** Chi Hong Sum, Nafiseh Nafissi, Roderick A. Slavcev, Shawn Wettig

**Affiliations:** 1 School of Pharmacy, University of Waterloo, 10 Victoria Street S., Kitchener, Ontario, Canada; 2 Waterloo Institute for Nanotechnology, University of Waterloo, 200 University Ave W., Waterloo, Ontario, Canada; University of Quebec at Trois-Rivieres, CANADA

## Abstract

In combination with novel **l**inear **c**ovalently **c**losed (LCC) DNA minivectors, referred to as DNA ministrings, a gemini surfactant-based synthetic vector for gene delivery has been shown to exhibit enhanced delivery and bioavailability while offering a heightened safety profile. Due to topological differences from conventional **c**ircular **c**ovalently **c**losed (CCC) plasmid DNA vectors, the linear topology of LCC DNA ministrings may present differences with regards to DNA interaction and the physicochemical properties influencing DNA-surfactant interactions in the formulation of lipoplexed particles. In this study, N,N-bis(dimethylhexadecyl)-α,ω-propanediammonium(16-3-16)gemini-based synthetic vectors, incorporating either CCC plasmid or LCC DNA ministrings, were characterized and compared with respect to particle size, zeta potential, DNA encapsulation, DNase sensitivity, and *in vitro* transgene delivery efficacy. Through comparative analysis, differences between CCC plasmid DNA and LCC DNA ministrings led to variations in the physical properties of the resulting lipoplexes after complexation with 16-3-16 gemini surfactants. Despite the size disparities between the plasmid DNA vectors (CCC) and DNA ministrings (LCC), differences in DNA topology resulted in the generation of lipoplexes of comparable particle sizes. The capacity for ministring (LCC) derived lipoplexes to undergo complete counterion release during lipoplex formation contributed to improved DNA encapsulation, protection from DNase degradation, and *in vitro* transgene delivery.

## Introduction

Gene therapy offers tremendous potential for the treatment of numerous diseases with demonstrated applications in vaccine development. Despite continuing successes of viral based gene therapeutics achieving significant clinical outcomes [1–4], these highly efficacious vectors present important safety concerns with respect to undesired immunostimulatory effects and/or insertional mutagenesis [5–8]. Furthermore, the application of viral vectors is hindered by limited repeat administrations due to pre-existing immunity, size of delivered gene construct, scale-up, as well as high production costs, contamination during production, and lack of desired tissue selectivity [5, 9]. Non-viral delivery vectors are generally advantageous over viral vectors with respect to safety, production costs, scalability, the ability to transfect larger sized DNA, and adaptability for different delivery options (e.g. targeted delivery, time-dependent release, enhanced circulation times, repeat administrations) [9, 10]. However, while preferential from a safety perspective, non-viral systems generally suffer associated low transfection efficiencies, an important obstacle that must be addressed in order for such systems to be recognized as effective vehicles for gene delivery.

**Fig 1:**
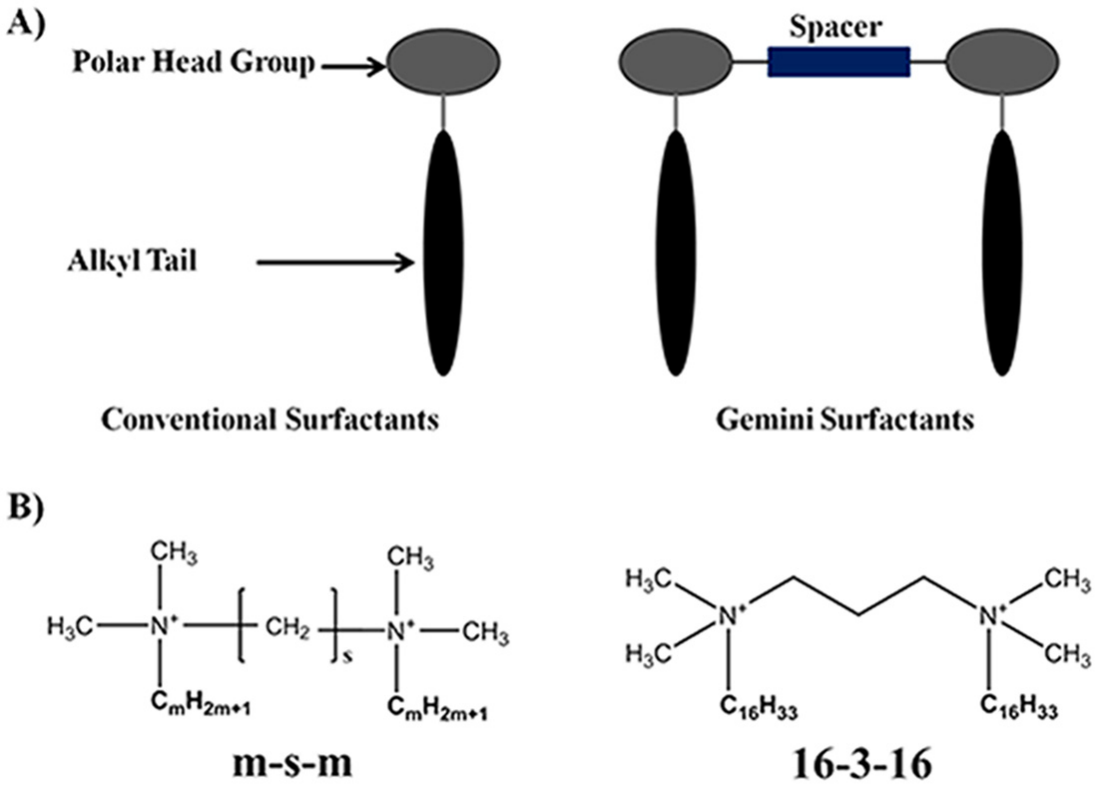
Structural schematic of conventional surfactants & gemini surfactants (A), and chemical structure of 16-3-16 gemini surfactant (B).16-3-16 Properties: cmc = 0.026 mM^32^, Krafft Temperature = 42°C ^39^.

Extensive efforts have been focused into the rational design of effective synthetic vectors with the capacity for DNA compaction and encapsulation, targeted delivery, cellular uptake and internalization, endosomal escape, and nuclear localization. Such efforts have culminated into the design and application of numerous cationic compounds as gene delivery vectors which contributed to the development of commercial cationic lipids, including Lipofectamine^™^ and Lipofectin^R^, suited for gene delivery. In consideration to the relatively high cost and short shelf-life associated with commercial vectors, cationic gemini surfactants have been synthesized as potential candidates for non-viral delivery. Gemini surfactants are amphiphilic molecules composed of two surfactant monomers (cationic, anionic, or neutral) chemically linked by a spacer (Fig 1). Gemini surfactants confer advantages of reduced cytotoxicity and cost effectiveness as they possess a critical micelle concentration (CMC) that is one to two orders of magnitude lower than their monomer counterparts [11–13]. Gemini surfactant derived synthetic vectors offer numerous advantages including: 1) high positive charge for effective DNA complexation at low concentrations; 2) efficient DNA compaction generating smaller complexes than their monomeric counterparts; 3) effective endosomal escape; and 4) suitability for long term storage in lyophilized formulations, over two months at ambient temperatures, without losing functionality [14, 15]. As such, different formulations of gemini surfactants, from traditional cationic m-s-m or N,N-bis(dimethylalkyl)-α,ω-alkanediammonium surfactants (where m and s represent the number of carbon atoms in the alkyl tails and the polymethylene spacer group) to peptide or carbohydrate based compounds, have been previously studied for applications in gene therapy [12].

Among the different m-s-m gemini surfactants, the 16-3-16 derivative has been extensively studied due to its structural nature, promoting effective DNA complexation, and its capacity to adopt structural polymorphisms critical to endosomal escape and successful gene delivery. The 16-3-16 gemini surfactant possesses a trimethylene spacer (s = 3) that provides compatible head group distances (~0.49 nm) with the spacing of phosphate groups (0.34 nm) in DNA [16]. The increased positive charge (relative to monomeric surfactants and lipids) promotes efficient DNA binding and compaction, generating particles suitable for gene delivery. Numerous reports have previously indicated the ability of 16-3-16 gemini-based lipoplexes, in combination with 1,2-dioleoyl-sn-glycero-3-phosphatidylethanolamine (DOPE) neutral lipid, to form higher ordered phase structures including inverted hexagonal and cubic phase structures [12, 17–19]. Such structures are highly dependent on the lipoplex composition with hexagonal structures predominantly present at high mol ratios of DOPE and cubic phase structures at high mol ratios of gemini surfactant [18]. The ability of such gemini-based lipoplexes to adopt structural polymorphisms is considered to be one of the most important factors contributing to improved gene delivery [9, 11, 12, 16, 18–20].

Highly efficacious gene therapeutics demand contributions from sound design of both the synthetic vector as well as the enclosed DNA cargo. Conventional recombinant plasmid DNA (pDNA) employed in non-viral gene delivery typically consists of two essential components: i) an eukaryotic expression cassette for the expression of the gene of interest, and ii) a prokaryotic backbone with an origin of replication for plasmid amplification and an antibiotic resistance gene cassette for selection [21]. While safer than their viral counterparts, non-viral delivery of such circular covalently closed (CCC) pDNA vectors, alone or packaged within synthetic vectors, offers a limited safety profile as they often result in the transfer of antibiotic resistance genes as well as other unwanted prokaryotic sequences with CpG motifs. The unnecessary delivery of antibiotic resistance genes may enable horizontal gene transfers, giving rise to antibiotic resistant pathogens. Unmethylated CpG dinucleotides, or CpG motifs, have the potential for eliciting immunostimulatory responses which reduce the efficacy of the gene therapeutic and may induce detrimental effects in the treated host [22–25]. Hence, the removal of the prokaryotic backbone in the generation of linear covalently closed (LCC) DNA minivectors serves the dual purpose of enhancing the safety of the delivered vector while improving the delivery process through the formation of smaller vectors that increase extracellular and intracellular bioavailability [21, 26].

LCC DNA minivectors are small, dumbbell shaped vectors possessing hairpin ends enclosing an eukaryotic expression cassette. The hairpin loops offer vast improvements in protection from exonucleases conferring greater stability, an issue that drastically hinders the successful delivery of linear DNA.LCC DNA vectors were shown to exhibit enhanced transgene expression over CCC pDNA counterparts as demonstrated by cytoplasmic and nuclear microinjections along with transfection using Lipofectamine^™^ [27–29]. In addition, LCC DNA minivectors offer a heightened safety profile as insertional mutagenesis is inhibited by the covalently closed terminal ends conferring double-strand breaks that cause chromosomal disruption and cell death in the low frequency event of chromosomal integration [26, 29].

**Fig 2:**
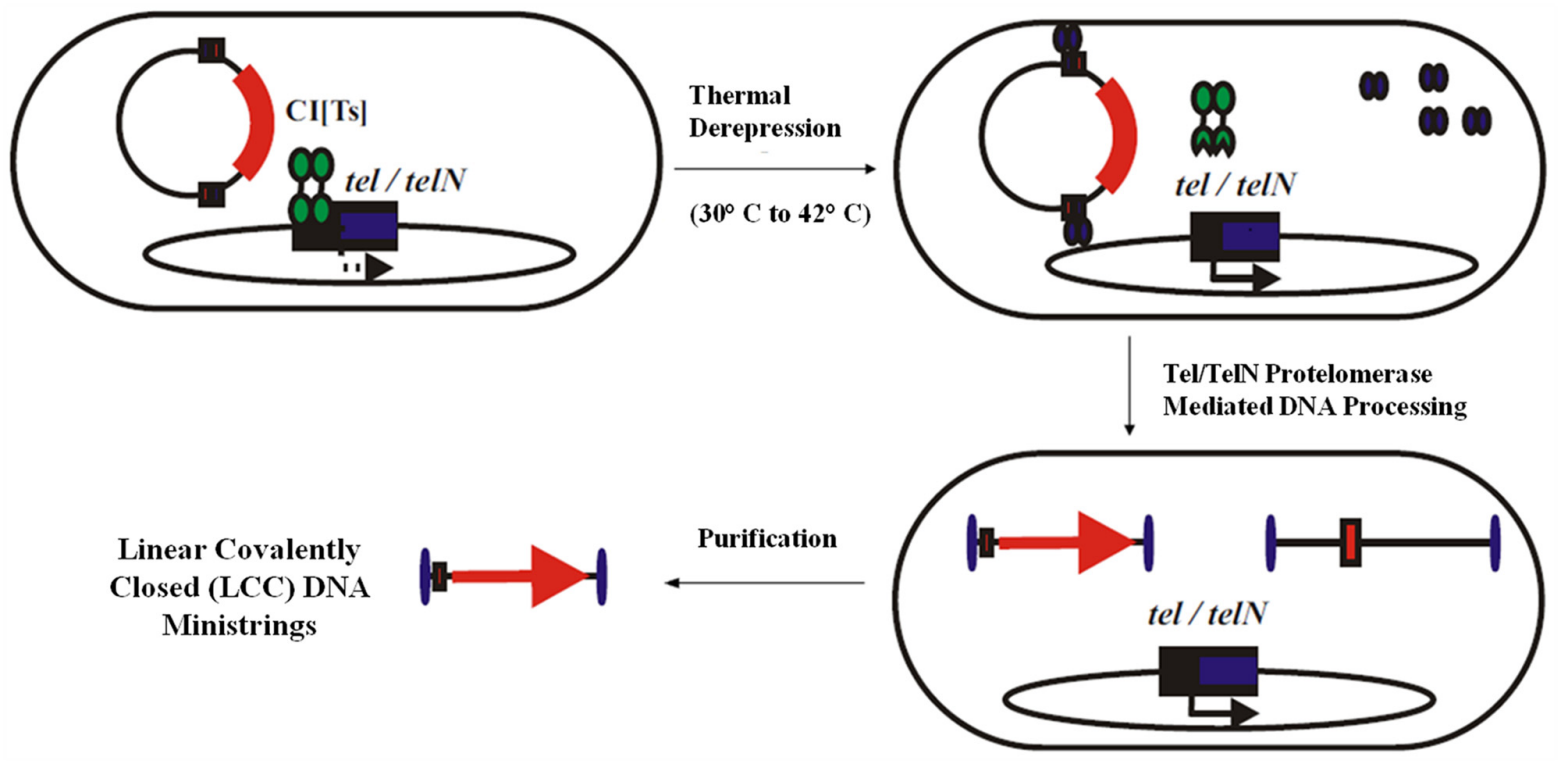
One-step *in vivo* LCC DNA minivector production system. The *in vivo* production system involves a recombinant *E*. *coli* for thermoregulated expression of Tel protelomerase. In the temperature inducible system, protelomerase expression is repressed by a CI[Ts]857 repressor at temperatures below 37°C. Temperature upshift to 42°C causes instability and dissociation of the thermolabile repressor which allows for controlled expression of protelomerase. Subsequent enzymatic activity of the expressed protelomerase on parental pDNA vector substrates results in DNA processing into LCC DNA ministrings.

**Fig 3:**
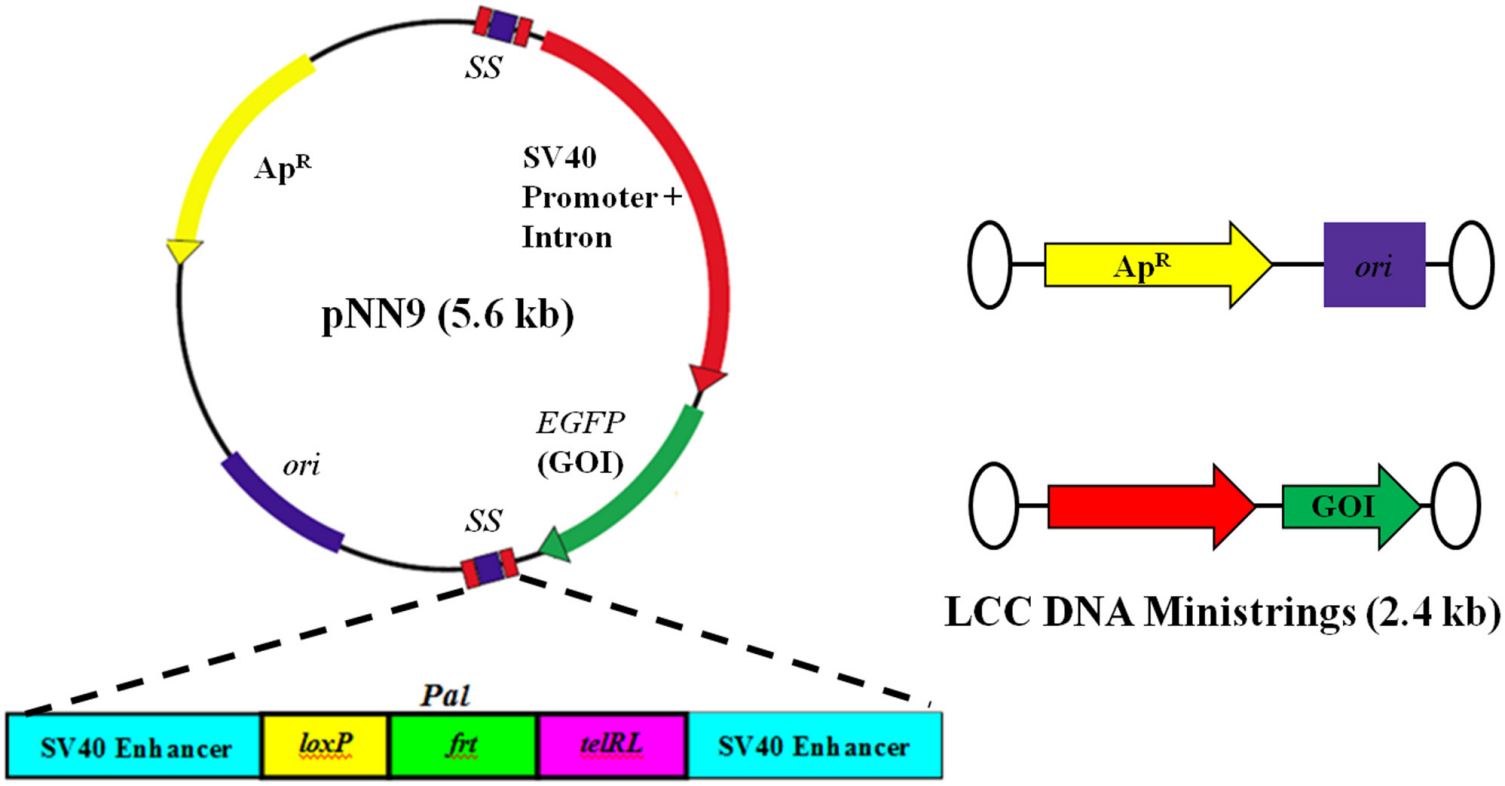
Parental plasmid DNA vector substrate for the production of LCC DNA ministrings. The 5.6 kb pDNA vector (pNN9) possesses two "Super Sequences" (SS) flanking the eukaryotic expression cassette for the generation of LCC DNA ministrings. Within each Super Sequence, *pal* act as the protelomerase recognition sequences for the production of LCC DNA ministrings (2.4 kb) upon processing by Tel protelomerases. SV40 enhancer sequences serve as DNA-targeting sequences (DTS) for improved nuclear entry during gene delivery.

We previously described an *E*. *coli* based one-step *in vivo* LCC DNA minivector production system for facile and efficient means of producing LCC DNA minivectors from parental CCC pDNA substrates (Fig 2) [26, 28]. The parental pDNA (Fig 3) is composed of an eukaryotic expression cassette flanked by two multi-target sites, called "Super Sequence" (SS), acting as recognition sites for PY54 bacteriophage derived Tel protelomerase. Temperature induced, *in vivo* expression of Tel protelomerases and their subsequent enzymatic activity, for excision and resolution of covalently closed terminal ends, result in the conversion of parental CCC pDNA into two smaller species: 1) a LCC backbone DNA carrying the unnecessary prokaryotic backbone, and 2) a LCC DNA minivector referred to as DNA ministrings [26]. Application of the novel *in vivo* DNA minivector production system permit the production of LCC DNA ministrings as well as the generation of safe and effective lipid-based synthetic vectors upon lipoplex formation with gemini surfactants.

Conventional gemini-based synthetic vectors for gene delivery generally consist of CCC pDNA vectors that differ in linear topology, DNA interactions, and physicochemical properties in comparison to LCC DNA ministrings. In light of these differences, we sought to characterize and compare the physical properties of the resulting lipoplexes after complexation with 16-3-16 gemini surfactants. Despite the size disparities between pDNA vectors (CCC) and DNA ministrings (LCC), differences in DNA topology resulted in the generation of lipoplexes of comparable particle sizes.

## Materials and Methods

### Strains and Plasmids

The pNN9 vector [26] was used as the parental pDNA substrate for the production of LCC DNA ministrings and for the generation of CCC pDNA derived lipoplexes. *E*. *coli* K-12 strains were used to generate all recombinant cell constructs and JM109 was employed as hosts for plasmid amplification.

### Production of CCC pDNA, LCC DNA Products and LCC DNA Ministrings

*E*. *coli* JM109 was used for amplification of the pNN9 parental vector (5.6 kb) (Table 1). A single colony of JM109[pNN9] was grown overnight in 5 ml Luria Bertani (LB) + ampicillin (Ap) (100 μg/ml) at 37°C with aeration. Four batches of fresh cells were subsequently grown overnight from the 5 ml culture at 1:100 dilution of 50 ml LB + Ap (100 μg/ml) in 250 ml Erlenmeyer flasks at 37°C with aeration. Cells were harvested and plasmid extracted with *E*.*Z*.*N*.*A*. Plasmid Maxi-Prep Kit (Omega, VWR). The extracted parental pDNA substrate was used for the generation of CCC/16-3-16 and CCC/16-3-16/DOPE lipoplexes.

**Table 1:** Generation of CCC pDNA and LCC DNA ministring-derived lipoplexes.

**DNA Construct**	**Nucleic Acid Size (Base Pair)**	**Molarity (pMol) per 1 μg**
pNN9 (CCC)	5621	0.28
DNA Ministring (LCC)	2410	0.62

The one-step *in vivo* LCC DNA minivector production system, Tel^+^W3NN[pNN9] *E*. *coli*, was used for the production of enhanced green fluorescent protein (*eGFP*) LCC DNA ministrings. A single colony of Tel^+^W3NN[pNN9] was grown overnight in 5 ml LB + Ap (100 μg/ml) under repressed conditions at 30°C with aeration. Two batches of fresh cells were grown from the overnight culture at 1:100 dilution of 50 ml LB + Ap (100 μg/ml) in 250 ml Erlenmeyer flasks at 30°C to late log phase A_600_ = 0.8. Cells were then collected, centrifuged at 4K RPM for 10 min, and re-suspended in 1 ml of LB + Ap. The re-suspensions were added into a preheated 2L Erlenmeyer flask containing 500 ml of LB + Ap (100 μg/ml) for incubation at 42°C until A_600_ = 1.0; followed by an additional 60 min incubation under the same conditions. Cultures were subjected to gradual temperature downshift and grown at 30°C overnight. Cells were harvested and plasmid extracted with *E*.*Z*.*N*.*A*. Plasmid Maxi-Prep Kit (Omega, VWR). The 2.4 kb LCC DNA ministrings were subsequently purified using agarose gel electrophoresis and gel extraction with *E*.*Z*.*N*.*A*. Gel Extraction Kit (Omega, VWR). The purified DNA ministrings were used for the generation of LCC/16-3-16 and LCC/16-3-16/DOPE lipoplexes.

### Generation of CCC pDNA and LCC Ministring-derived Lipoplexes

16-3-16 gemini surfactants were previously synthesized according to procedures outlined by Wettig and Verrall [30]. 1.5 mM 16-3-16 gemini surfactant stock solutions were prepared in molecular water (HyClone, Thermo Scientific) with sonication (50°C) and purification through a 0.2 μm sterile filter (Fisher Scientific, Canada). Different aliquots of the 16-3-16 stock solution (1.2 μl, 3 μl and 6 μl per 0.4 μg DNA) were used to generate DNA/16-3-16lipoplexes at 2:1, 5:1, and 10:1 N^+^/P^−^ charge ratios.1 mM DOPE vesicles were prepared in phosphate buffer saline (PBS) according to procedures outlined by Wettig et al. [20]. The vesicles were filtered through a 0.45 μm sterile filter and different aliquots (3 μl, 7.4 μl, and 15 μl) were used to generate DNA/16-3-16/DOPE lipoplexes, of varying charge ratios, with a constant gemini to DOPE ratio of 1:2.5.

The two respective lipoplexes were prepared as follows: 0.4 μg of DNA, pNN9 (5.6 kb) or *eGFP* LCC DNA ministring (2.4 kb), was mixed with different aliquots of 1.5 mM 16-3-16 gemini surfactant solution to yield N^+/^P^−^ charge ratios of 2:1, 5:1, and 10:1 (Table 1). After 15 min incubation at room temperature, different aliquots of 1 mM DOPE were added and the subsequent mixture was further incubated for 30 minutes at room temperature. All other DNA/16-3-16/DOPE lipoplexes of different N^+^/P^−^ charge ratios were generated in the same manner using different aliquots/dilutions of 16-3-16 gemini and DOPE.

### Characterization of 16-3-16 Gemini-based Lipoplexes

#### Particle Size and Zeta Potential

Particle sizes for DNA, 16-3-16, DOPE, and resulting DNA/16-3-16 & DNA/16-3-16/DOPE lipoplexes were measured by dynamic light scattering using a Malvern Zetasizer Nano ZS instrument (Malvern instruments, UK). Particle size distributions were obtained from light scattering (θ = 173°) in water at 25°C and the measured sizes were reported using a percent volume distribution. Samples were measured in triplicates of triplicate and the resulting averages were reported.

Zeta potential (ζ) for the abovementioned samples was measured by Laser Doppler Electrophoresis using zeta potential capillary cells and a Malvern Zetasizer Nano ZS instrument (Malvern instruments, UK). All measurements were made at 25°C and samples were measured in triplicates of quintuplicate with averages being reported.

#### DNase Sensitivity Assay

The DNase sensitivity assay involved the incubation of lipoplexes with DNase I (1 unit per 1 μg DNA) (Promega) and the DNase reaction buffer (Tris-HCl, M_g_SO_4_, CaCl_2_) for 30 minutes at 37°C. Subsequently, DNase I was inactivated by the addition of DNase stop solution (ethylene glycol tetra acetic acid (EGTA)) and denatured upon 10 minute incubation at 60°C. Lipoplexes were disrupted with the addition of phenol:chloroform:isoamyl alcohol (25:24:1, v/v) (Invitrogen) for the recovery of non-degraded DNA upon centrifugation. The extent of DNase I induced degradation was assessed by agarose gel electrophoresis upon equal loading across all samples.

#### In vitro Transgene Delivery Assay

Human-derived ovarian cancer cells, OVCAR-3 (Invitrogen) were grown in RPMI+ GlutaMAX supplemented with 20% fetal bovine serum, 100 μg/ml streptomycin, and 100 IU/ml penicillin. All cell culture reagents and cell culture equipment were provided by Life Technologies (Carlsbad, CA) and VWR (Radnor, PA), respectively. Cationic lipid transfection reagents Lipofectamine^™^ LTX, and Plus reagents were obtained from Invitrogen. To transfect cells, 5.0 × 10^5^ OVCAR-3 cells were seeded into 24-well culture plates 24 h before transfection in complete media without antibiotic. One hour prior to transfection, the culture medium was replaced with serum-free RPMI medium. 0.4 μg of DNA (pNN9 or DNA ministring), diluted in 50 μl of serum-free OptiMEM culture medium, was mixed with different aliquots of 1.5 mM 16-3-16 gemini surfactant solution to yield N^+^/P^−^ charge ratios of 3:1 and 5:1. After 15 min incubation at room temperature, appropriate aliquots of 1 mM DOPE were incorporated to achieve a constant gemini to DOPE ratio of 1:2.5. The subsequent complexes were further incubated for 30 minutes at room temperature. Cationic complexes of Lipofectamine^™^ LTX were prepared according to manufacturer's protocol with no deviation. The mixture of pNN9/16-3-16 & pNN9/16-3-16/DOPE and DNA ministring/16-3-16 &DNA ministring /16-3-16/DOPE complexes was added drop-wise to each well (duplicate). The plate was centrifuged for 5 min at 200 RPM, at room temperature, prior to incubation at 37°C. At 5 hours post transfection, the transfected media was replaced with fresh complete media free of antibiotic. Transfection efficiency was assessed by flow cytometry after subsequent 48 h incubation at 37°C.

#### Flow Cytometry

Transfection efficiency was determined 48 h after transfection by flow cytometry. Cells were trypsinized, washed with PBS, and counted. Data were collected from 10^4^ events. Cells were stained by ten microliters of the cell membrane impermeable, intercalating red fluorescent propidium iodide (PI), 20 mg/ml Sigma-Aldrich (St Louis, MO), to measure cytotoxicity after transfection by excluding dead cells from viable cells. Untreated cells served as controls for cytotoxicity and GFP expression. GFP expression levels were calculated by multiplying the mean relative fluorescence values of transfected cells by the percentage of transfected cells. This parameter is considered to be directly proportional to the total amount of produced transgene product. All data were expressed by GraphPad as mean ±SEM. Statistical differences were determined using a two-way ANOVA test followed by Bonferroni's post-tests. Significance was set at a p<0.05.

## Results

### Particle Size and Zeta Potential (ζ)

With regards to 5.6 kb pNN9 (CCC) and 2.4 kb DNA ministring (LCC), particle sizes of the two DNA vectors were surprisingly similar despite their inherent differences in DNA composition (Table 2). The differences were accounted by the supercoiled nature of CCC pDNA contributing to more compact conformations than their linear counterparts. Such notions were supported by the differences in observed zeta potentials (ζ) as DNA supercoiling in pNN9 (CCC) masked a fraction of the negative charges attained from the phosphate groups of DNA. In contrast, the structural nature of the linear isoforms contributed to more prominent surface charges as indicated by a greater (-) zeta potential. By itself, 16-3-16 gemini surfactants were able to rapidly self-assemble into micelles due to high concentrations of the stock solution and due to conditions at which lipoplexes were generated. The 1.5 mM 16-3-16 stock solution was generated at concentrations well above the CMC of the gemini surfactant (0.0255 mM) and as lipoplexes were generated below the Krafft temperature (*T*_*k*_ = 42°C), referred to as the minimum temperature at which surfactant forms micelles, 16-3-16 micelles were rapidly self-assembled [16, 31]. The propensity for 16-3-16 gemini surfactant to form micelles/vesicles of varying sizes resulted in the observed high polydispersities as indicated by a PDI value of 0.752.

**Table 2:** Particle size & zeta potential of CCC pNN9, LCC DNA ministring, 16-3-16, and DOPE.

	Size (d.nm)	Polydispersity Index (PDI)	ζ-Potential (mV)
pNN9 (CCC)	371 ± 90	0.466	-25 ± 9
DNA ministring (LCC)	363 ± 74	0.494	-35 ± 2
16-3-16	50 ± 17	0.752	55 ± 5
DOPE	131 ± 18	0.257	-19 ± 5
16-3-16/DOPE	204 ± 67	0.657	

**Fig 4:**
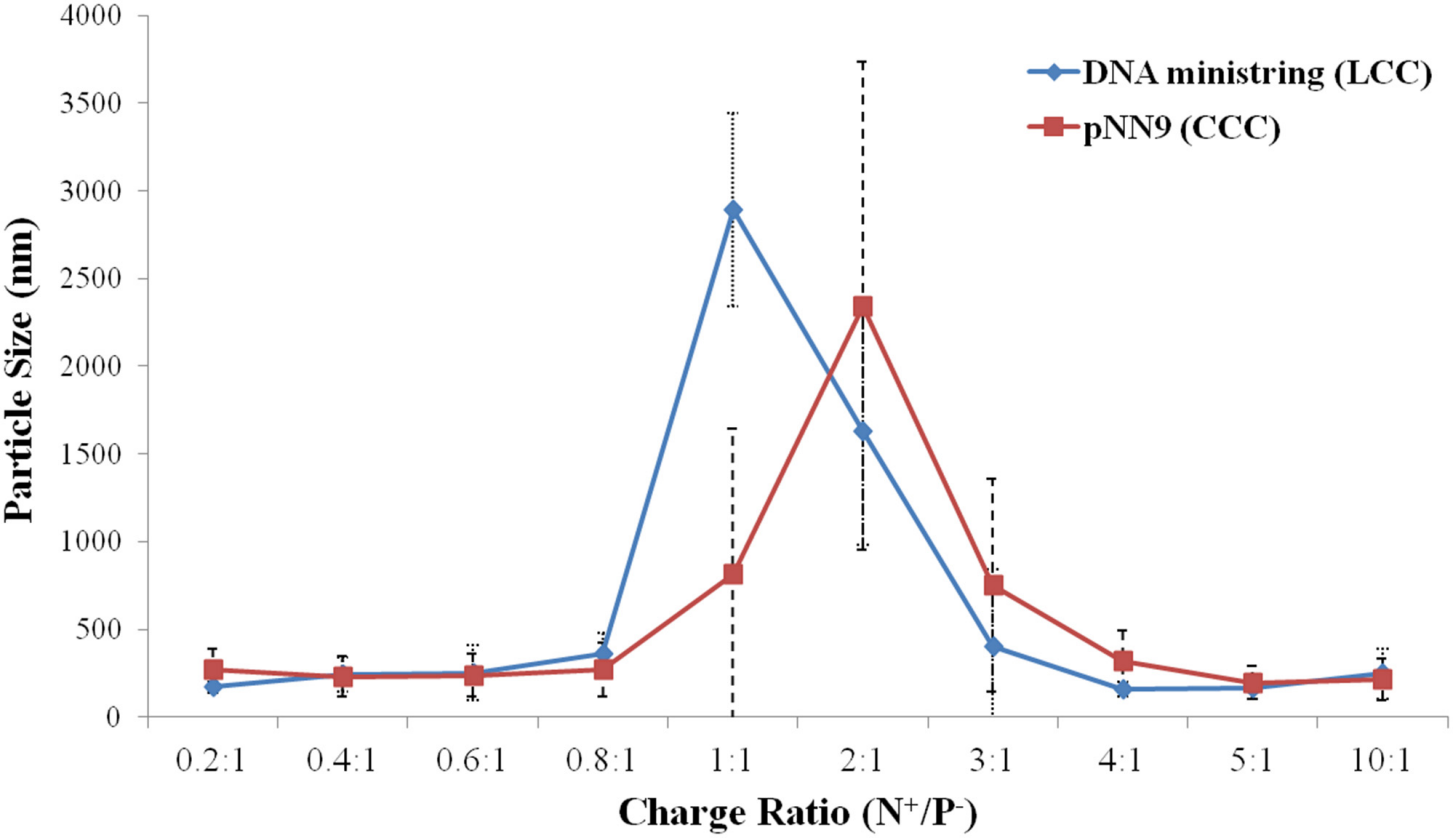
Particle size variations for DNA/16-3-16/DOPE lipoplexes with increasing N^+^/P^−^ charge ratios. As lipoplexes approach charge neutralization, a significant increase in particle size led to highly variable particles conferring large aggregate formation. Large aggregates for LCC/16-3-16/DOPE and CCC/16-3-16/DOPE lipoplexes appeared most prominent at charge ratios of 1:1 and 2:1 respectively. Progressive decreases to particle sizes at higher charge ratios led to stable and uniform particle formation.

With respect to DNA/16-3-16 and DNA/16-3-16/DOPE lipoplexes, the two vectors generated lipoplexes of comparable particle sizes and zeta potentials across the three tested charge ratios (Table 3). For DNA/16-3-16 lipoplexes, both CCC/16-3-16 and LCC/16-3-16 lipoplexes exhibited the progressive formation of uniformly sized particles, as indicated by decreasing PDI values, at increasing charge ratios. With respect to DNA/16-3-16/DOPE lipoplexes, substantially larger particle sizes were observed for lipoplexes at charge ratios of 2:1. The large particles exhibited at lower charge ratios were likely the result of aggregation upon charge neutralization and subsequent addition of more gemini surfactant, at 5:1 and 10:1 charge ratios, resulted in a dramatic decrease in particle sizes with lower polydispersities. In closer inspection of particle size fluctuations across the spectrum of progressively increasing charge ratios (Fig 4), charge neutralization and aggregation for LCC/16-3-16/DOPE lipoplexes was observed to occur at the lower charge ratio of 1:1 whereas significant aggregation of CCC/16-3-16/DOPE lipoplexes occurred at a charge ratio of 2:1. Aggregation of the resulting lipoplexes and interference with light scattering measurements, upon charge neutralization, contributed to large standard deviations and populations of highly variable particle sizes [32, 33].

**Table 3:** Particle size and zeta potential of DNA/16-3-16 and DNA/16-3-16/DOPE lipoplexes.

	pNN9 (CCC)			DNA ministring (LCC)		
	Size (d.nm)	PDI	ζ-Potential (mV)	Size (d.nm)	PDI	ζ-Potential (mV)
**DNA/16-3-16**						
2:1	141 ± 24	0.402	38 ± 1	143 ± 40	0.351	41 ± 9
5:1	127 ± 31	0.359	39 ± 3	114 ± 37	0.241	48 ± 7
10:1	100 ± 38	0.267	48 ± 9	132 ± 43	0.185	43 ± 3
**DNA/16-3-16/DOPE**						
2:1	2343 ± 1390	0.592	10 ± 18	1634 ± 655	0.561	22 ± 3
5:1	195 ± 95	0.346	24 ± 7	168 ± 65	0.243	29 ± 7
10:1	218 ± 119	0.372	36 ± 6	247 ± 144	0.409	29 ± 6

#### DNase Sensitivity

**Fig 5:**
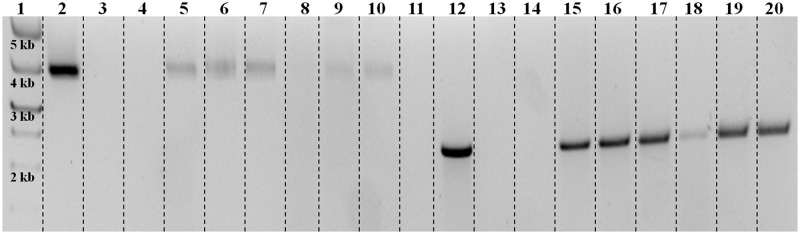
DNase sensitivity assay for DNA/16-3-16 and DNA/16-3-16/DOPE lipoplexes. DNA ladder (lane 1); pNN9 (CCC) (lane 2); DNase I exposed pNN9 (CCC) (lanes 3 & 4); CCC/16-3-16 lipoplexes at 2:1, 5:1, and 10:1 charge ratios (lanes 5–7); CCC/16-3-16/DOPE lipoplexes at 2:1, 5:1, and 10:1 charge ratios (lanes 8–10); 16-3-16/DOPE (lane 11); DNA ministring (LCC) (lane 12); DNase I exposed DNA ministring (LCC) (lanes 13 & 14); LCC/16-3-16 lipoplexes at 2:1, 5:1, and 10:1 charge ratios (lanes 15–17); LCC/16-3-16/DOPE lipoplexes at 2:1, 5:1, and 10:1 charge ratios (lanes 18–20). Equal loading of the recovered DNA solution after DNase I exposure indicated improved DNA recovery for ministring (LCC) derived lipoplexes. DNA/16-3-16 lipoplexes conferred better DNA recovery after DNase I degradation over DNA/16-3-16/DOPE lipoplexes across all of the tested charge ratios.

**Fig 6:**
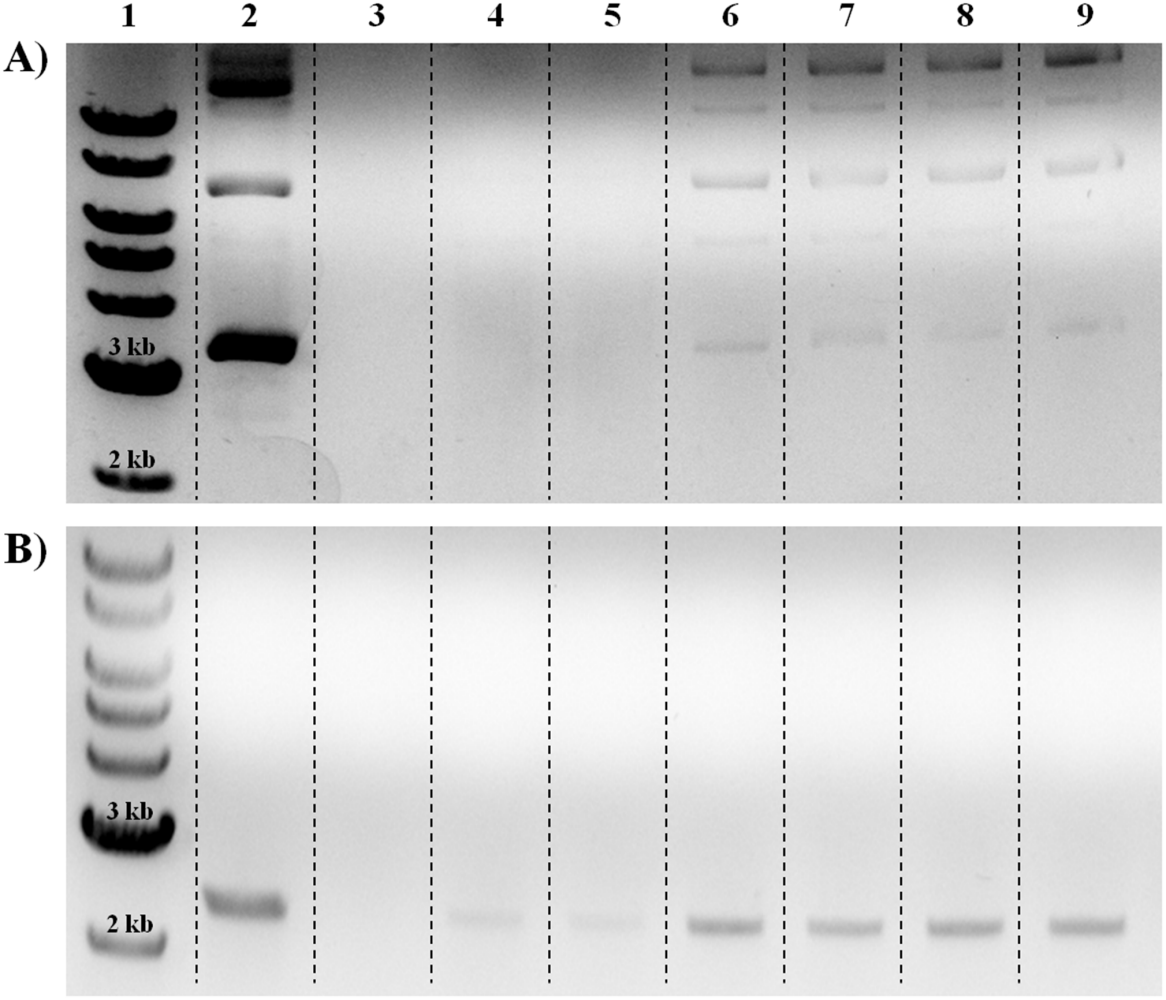
DNase sensitivity assay for A) CCC/16-3-16/DOPE lipoplexes and B) LCC/16-3-16/DOPE lipoplexes. DNA ladder (lane 1); DNA (lane 2); DNase I exposed DNA (lane 3); DNA/16-3-16/DOPE lipoplexes at 2:1 charge ratio (lanes 4 & 5); DNA/16-3-16/DOPE lipoplexes at 5:1 charge ratio (lanes 6 & 7); DNA/16-3-/16/DOPE lipoplexes at 10:1 charge ratio (lanes 8 & 9). Equal loading confirmed improved DNA recovery after DNase I exposure at higher charge ratios. A majority of the complexed DNA ministrings (LCC) was recovered in lipoplexes at 5:1 and 10:1 charge ratios.

Results from the DNase sensitivity assay show improved DNA protection in DNA ministring (LCC) derived lipoplexes (Fig 5). Lipoplexes formed in absence of DOPE offered better protection as indicated by improved DNA recovery. Across all three tested N^+^/P^−^ charge ratios, DNA ministring (LCC) derived lipoplexes (lanes 15–17) exhibited improved stabilities over pNN9 (CCC) derived lipoplexes (lanes 5–7) as evidenced by greater DNA recovery upon DNase exposure. With respect to LCC/16-3-16 and LCC/16-3-16/DOPE lipoplexes, the incorporation of DOPE resulted in less stable complexes as limited amounts of DNA were recovered for LCC/16-3-16/DOPE lipoplexes at 2:1 charge ratio (lane 18); however, greater stabilities and improved DNA recovery were observed at higher charge ratios for DOPE containing lipoplexes (lanes 19 & 20). Comparative analysis between LCC/16-3-16/DOPE and CCC/16-3-16/DOPE lipoplexes, at 2:1, 5:1, and 10:1 charge ratios, confirmed improved DNA protection for LCC/16-3-16/DOPE lipoplexes (Fig 6). As a majority of the complexed DNA was protected from DNase I degradation, LCC/16-3-16/DOPE lipoplexes at 5:1 and 10:1 charge ratios may serve to be suitable candidates as synthetic vectors.

#### DNA ministrings exhibit improved transfection efficiency

**Fig 7:**
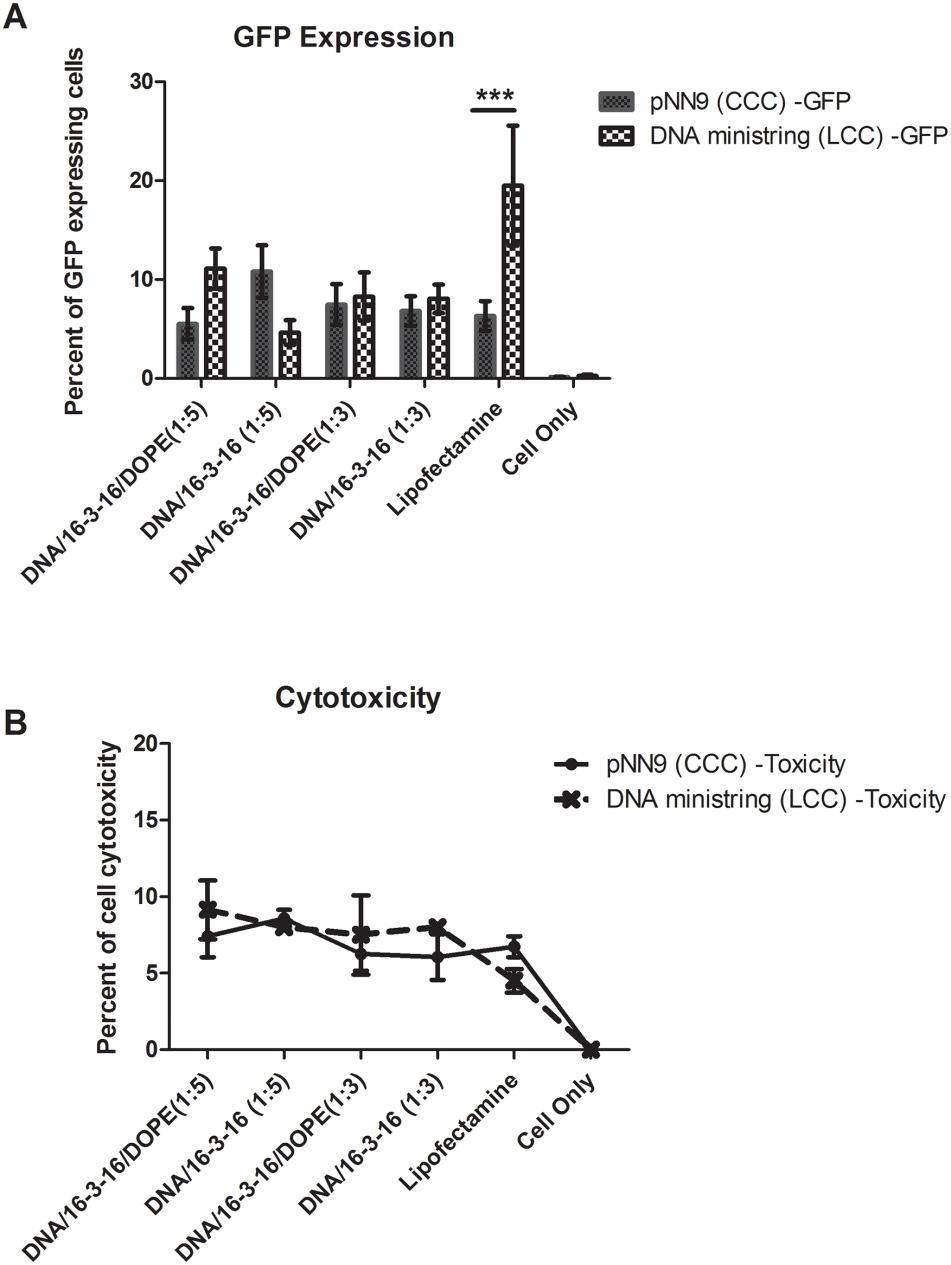
Effect of DNA topology on *in vitro* gemini surfactant-based transgene delivery. Parent CCC plasmid (pNN9) DNA vectors were processed into LCC DNA ministrings by passing through the one-step heat-inducible mini DNA vector production system. 0.4 μg of each respective DNA vector was mixed with gemini surfactant 16-3-16 at charge ratios of 5:1 or 3:1 in presence or absence of helper lipid, DOPE, and transfected into human-derived ovarian cancer (OVCAR-3) cells. Cells were collected 48 h post-transfection and analyzed by flow cytometry for green fluorescent protein (eGFP) expression (A) and cytotoxicity of synthetic carriers using red propidium iodide (PI) fluorescent protein (B). Transfection efficiency was measured as the number of eGFP-expressing cells divided by the total number of cells. PI was added to assess transfection associated cytotoxicity. All data were expressed by GraphPad as mean ±SEM. Statistical differences were determined using a two-way ANOVA test followed by Bonferroni's post-tests. Stars indicate significantly higher transfection efficiencies of LCC DNA ministrings compared to pNN9 parent plasmid (*P* ≤ 0.001).

We previously constructed a pGL2 (Promega, Madison, WI) vector derivative that expressed enhanced green fluorescent protein (*eGFP*) under the control of an SV40 promoter and two specially designed target sequences of the Tel protelomerase referred to as the super sequence (SS) (Fig 3). The derivative LCC DNA ministring was generated from parent CCC plasmid using a one-step heat-inducible mini DNA vector production system as previously described [28]. Transfection complexes were prepared at the DNA/16-3-16 at charge ratios of 3:1 and 5:1, with and without DOPE, in accordance to the particle size and DNase sensitivity results. Direct comparison with equal amounts (by weight) of LCC DNA ministrings and conventional CCC parent plasmids indicated no statistically significant differences with respect to gemini-mediated transfection efficiencies (Fig 7-A). However, transfection using Lipofectamine^™^ indicated that LCC DNA ministrings imparted significantly higher transfection efficiency than their parental CCC counterparts (*P* < 0.001). No statistically significant differences were observed with respect to gemini-mediated cytotoxicity, in presence or absence of helper lipid DOPE, between LCC DNA ministring and CCC pNN9 derived lipoplexes (Fig 7-B).

## Discussion

With respect to the influences of DNA topology, direct comparisons between CCC pNN9 and LCC DNA ministrings cannot be made due to the inherent differences in the size of the two respective DNA vectors. More apparent differences may have arisen had the parental supercoiled CCC pNN9 been compared with its parental LCC counterpart, however, results from this study denoted certain differences arising from the influences of DNA topological conformations. The supercoiling effect contributed to a lower effective negative charge for CCC pNN9 [34, 35] which led to lower surface charges (ζ = -25 ± 9 mV) when compared to LCC DNA ministrings (ζ = -35 ± 2 mV). In addition, DNA supercoiling reduced the overall size of the circular plasmid, which contributed to comparable particle sizes between pNN9 and DNA ministrings despite the fact that pNN9 was the larger sized plasmid. Such differences had significant effects on the interactions between DNA and 16-3-16 gemini surfactant in terms of counterion release during lipoplex formation. Previously, DNA/16-3-16/DOPE lipoplexes, comprised of linear calf thymus DNA (ctDNA), demonstrated complete release of Na^+^ counterions during lipoplex formation; in contrast, a significant fraction of counterions remained bound during complex formation of CCC pDNA-derived lipoplexes [34, 35]. Counterion displacement was suggested to be inhibited due to the compact conformation of supercoiled CCC pDNA inducing geometric constraints on gemini/DNA interactions.

Resulting particle sizes and zeta potentials for DNA/16-3-16 and DNA/16-3-16/DOPE lipoplexes were in agreement with literature [19, 36] as all lipoplexes possessed positive zeta potentials critical to *in vitro* transfection. Upon inspection of particle size variations for lipoplexes across different charge ratios, both CCC/16-3-16/DOPE and LCC/16-3-16/DOPE exhibited significant increases in particle sizes at charge ratios corresponding to charge neutralization and large aggregate formation. For CCC/16-3-16/DOPE lipoplexes, substantial large aggregation formation was observed at a higher charge ratio of 2:1 in contrast to 1:1 for LCC/16-3-16/DOPE lipoplexes. Differences may be attributed to the antagonistic interactions between 16-3-16 gemini and DOPE [37] in combination with incomplete counterion release for CCC/16-3-16 lipoplexes, prompting more prominent DOPE induced instabilities that prevented the generation of stable, discrete lipoplex particles. Lipoplex instabilities were exemplified by the lower and highly variable (+) zeta potentials (ζ = 10 ± 18 mV) for CCC/16-3-16/DOPE lipoplexes at a charge ratio of 2:1. Such zeta potentials were indicative of charge neutrality that contributed to aggregation and the observed large particle sizes.

DNA ministring (LCC) derived lipoplexes exhibited improved DNA encapsulation and protection properties as evidenced by improved DNA recovery upon DNase I exposure. For both CCC/16-3-16/DOPE and LCC/16-3-16/DOPE lipoplexes, the higher charge ratios of 5:1 and 10:1 elicited better DNA encapsulation, protecting the DNA cargo from degradation. However, such protection was more prominent in LCC/16-3-16/DOPE lipoplexes and this was attributed to the higher (-) zeta potential of DNA ministrings and the complete release of counterions during complexation. The highly negative zeta potentials exhibited in DNA ministrings denoted significant surface charges for extensive electrostatic interaction with the positively charged 16-3-16 gemini surfactant, leading to complete counterion release and reduced head group repulsions. Reduced head group repulsion between individual gemini surfactants conferred better encapsulation, effectively protecting the residing DNA from exposure to DNaseI. With regards to LCC/16-3-16 and LCC/16-3-16/DOPE lipoplexes, improved DNA encapsulation and protection for LCC/16-3-16 lipoplexes were attributed to tight associations between DNA ministring and gemini surfactant as supported by high (+) zeta potentials [18].

## Conclusion

The differences in topology between conventional CCC pDNA vectors and LCC DNA ministrings influenced the complexation of gemini surfactants during lipoplex formation and the generation of lipid-based synthetic vectors. Such differences contributed to variations in particle size as well as the capacity for effective DNA encapsulation and protection from DNase I degradation. Further investigation, through additional physical characterization (e.g. isothermal titration calorimetry (ITC) & small angle X-ray scattering (SAXS)), will be warranted to fully ascertain the influences of DNA topology on transfection capacities of gemini-based synthetic vectors.
